# Innovative Methods in Chronic Disease: Advancing Qualitative, Intersectional, and Community-Based Strategies

**DOI:** 10.1093/geroni/igaf122.1733

**Published:** 2025-12-31

**Authors:** Weiyu Mao, Hanzhang Xu, Bei Wu

## Abstract

Despite the growing diversity in older Americans, engaging older adults from underrepresented communities in research remains challenging. This presents opportunities for enhancing diversity within aging research through innovative methodological approaches. To address significant disparities in representation, this symposium collectively investigated innovative methodologies that promote inclusivity, diversity, and engagement involving qualitative, intersectional, and community-based strategies. The first study utilized reflexive thematic analysis of three qualitative studies among Chinese Americans on different health topics. It recognized patterns of participant responses that lacked depth from qualitative interviews and discussed practical strategies and important considerations to amplify diverse participant voices, thereby preventing or reducing potential biases in data collection and analysis. The second study compared methodology and recruitment strategies in a qualitative study and a randomized controlled trial on Chinese and Korean American dementia caregivers. It highlighted the importance of understanding the influences of methodology on recruitment strategies and participant characteristics. The third study employed an intersectionality framework to analyze cardiovascular-kidney-metabolic syndrome stages and associated risks in the All of Us program. Findings from this study demonstrated varied risks by ethnic and racial designation, gender, and disadvantaged area of residence. The fourth study contrasted recruitment strategies of Chinese Americans with chronic conditions through electronic health records versus community engagement. The findings suggested differing participant characteristics by recruitment methods and highlighted the need for complementary approaches to ensure representativeness in clinical research. Together, these studies offered unique insights into advancing innovative research methods and highlighting practical implications on recruitment, engagement, and representation for vulnerable populations.

